# Pre-mRNA Splicing Functions in Plant Sexual Reproduction Development

**DOI:** 10.3390/plants14101472

**Published:** 2025-05-14

**Authors:** Dongjie Shao, Xinqi Gao, Yiming Wei

**Affiliations:** 1 College of Life Sciences, Zaozhuang University, Zaozhuang 277160, China; shaodongjie@uzz.edu.cn; 2 State Key Laboratory of Wheat Improvement, College of Life Sciences, Shandong Agricultural University, Taian 271018, China

**Keywords:** gametophyte formation, embryo development, pre-mRNA splicing, plant sexual reproduction

## Abstract

Precursor messenger RNA (pre-mRNA) splicing is a critical post-transcriptional regulatory mechanism in gene expression. The precise splicing of pre-mRNAs is essential for plant development and responding to genetic and environmental signals. In plant sexual reproduction, gene expression regulation relies on the accurate processing of pre-mRNAs, which is fundamental for coordinating developmental programs. The alternation of generations in plants involves two key phases: gametophyte development, which produces gametes, and fertilization, which leads to the formation of a diploid sporophyte. Gametophyte and embryo development represent essential processes in plant sexual reproduction. This review focuses on summarizing and analyzing the current evidence regarding the role of pre-mRNA splicing in plant sexual reproduction, with an emphasis on its involvement in gametophyte formation and embryo development. Future challenges in understanding RNA splicing regulation in plant sexual reproduction are also discussed, particularly in modulating splicing factor levels and activities and identifying target mRNAs and non-coding RNAs regulated by these factors. This review provides crucial insights into the molecular mechanisms of plant reproductive development and offers a theoretical basis for improving plant fertility and adaptability via RNA splicing regulation.

## 1. Introduction

Higher plants undergo two distinct multicellular stages in their life cycle: the haploid gametophyte (pollen and embryo sac) and the diploid sporophyte (embryo and seedling). Gametophytes generate gametes, and their fusion gives rise to the diploid sporophyte, marking two critical phases in the alternation of generations. Gene expression regulation plays a key role in gametophyte formation and embryo development. Precursor messenger RNA (pre-mRNA) splicing is a post-transcriptional regulatory mechanism that involves the removal of introns and the joining of exons to produce mature mRNAs for translation. Since plant growth and development rely on precise gene expression, accurate pre-mRNA processing is essential for maintaining normal cellular functions and responding to genetic and environmental signals [1,2,3,4,5,6]. Studies have shown that pre-mRNA splicing influences plant sexual reproduction. It is noteworthy that many genes involved in plant pre-mRNA splicing are essential genes, and their homozygous mutations often result in embryonic lethality, while defects in heterozygous mutants are particularly pronounced in the haploid gametophyte and early embryogenesis. Therefore, the haploid generation and early embryogenesis stage are ideal for analyzing the role of pre-mRNA splicing in plant sexual reproduction. This review summarizes the current findings on the role of pre-mRNA splicing in gametophyte formation and embryo development and discusses future challenges in understanding its regulatory impact on plant reproduction.

## 2. Pre-mRNA Splicing

**Figure 1:**
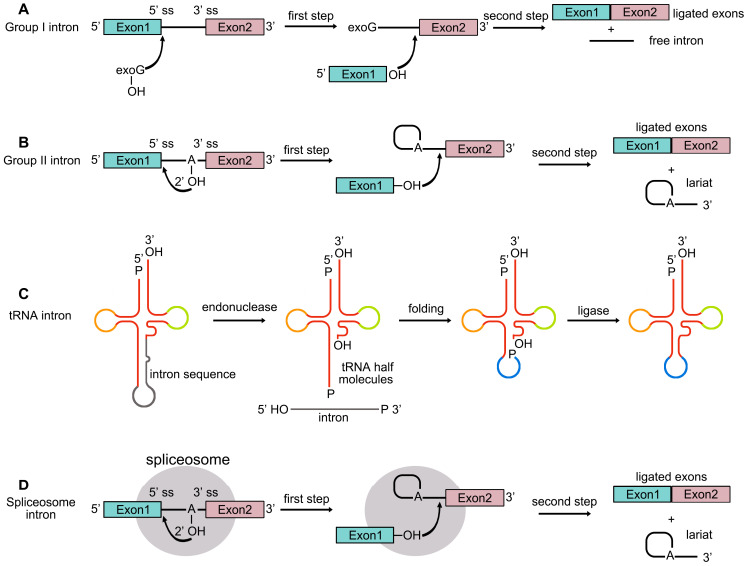
Schematic representation of four pre-mRNA splicing mechanisms. (**A**) In group I introns, splicing is initiated when a cofactor attacks the 5′ splice site (5′ ss) and attaches to the intron, releasing the upstream exon. The 3′ end of the released exon then attacks the 3′ splice site (3′ ss), resulting in exon ligation and intron release. (**B**) Group II introns undergo a similar process, where the 2′-OH of adenosine at the branch site attacks the 5′ ss, leading to cleavage of the 5′ exon and formation of a lariat-3′exon intermediate. The 3′-OH of the cleaved 5′ exon then attacks the 3′ ss, producing ligated exons and a lariat intron. (**C**) tRNA introns are removed by a splicing endonuclease, generating exons with 2′,3′-cyclic phosphate and 5′-OH ends. The exon halves fold into a tRNA-like structure, which is then sealed by a ligase. (**D**) Spliceosome introns follow a two-step transesterification reaction akin to group II introns, but the process is catalyzed by the spliceosome complex.

Introns are non-coding sequences within genes that are removed from precursor RNA during splicing. Based on their splicing mechanisms, introns are classified into four major groups: self-splicing group I and group II introns, tRNA and/or archaeal introns, and spliceosome-catalyzed introns [7,8,9,10]. The excision of group I introns occurs through a two-step transesterification reaction that requires exogenous guanosine (exoG) as a cofactor (Figure 1A). On the other hand, group II introns undergo splicing via a different mechanism, forming a lariat structure during intron removal (Figure 1B). The removal of introns from tRNA involves splicing endonuclease and ligase, which process the precursor to generate a functional tRNA (Figure 1C). Spliceosomal introns in nuclear pre-mRNA are excised by the spliceosome, a large ribonucleoprotein complex [11], and their removal follows a mechanism similar to that of group II introns (Figure 1D).

**Figure 2:**
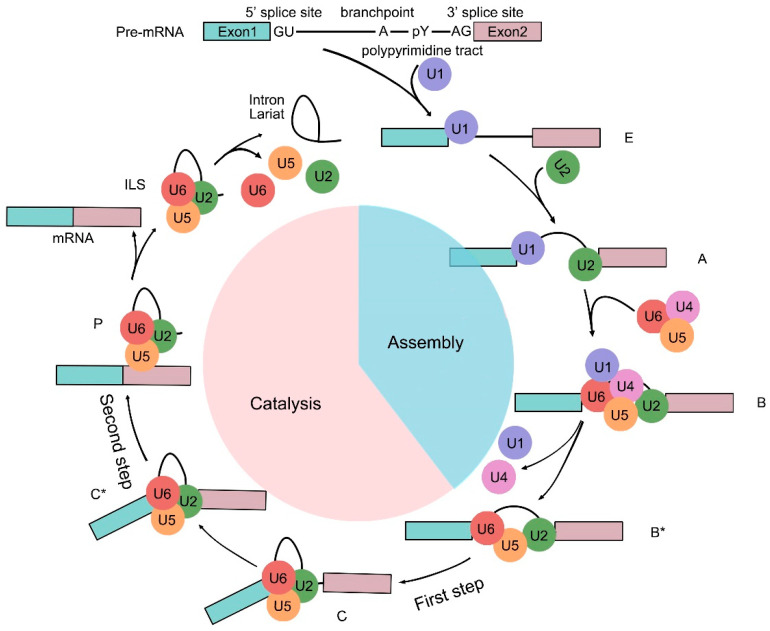
A basic model of the RNA splicing mechanism. The schematic diagram represents the spliceosome assembly and catalysis during transcript maturation. The process begins with U1 snRNP binding to the 5′ splice site of the intron in the pre-mRNA, forming complex E, while U2 snRNP binds to the branch site to form complex A. The U4/U5/U6 tri-snRNP then joins complex A to form the B complex. After the dissociation of U1 and U4, the spliceosome undergoes rearrangements, resulting in the activated Bact complex. The catalytically active B* complex is formed, facilitating the two transesterification reactions that remove the intron lariat and ligate the two exons. E: early complex, the early spliceosome. A: A complex, pre-spliceosome. B: B complex, precatalytic spliceosome. B*: B*complex, catalytically activated spliceosome. C: C complex, catalytic step I spliceosome. C*: C* complex, step Ⅱ catalytically activated spliceosome. P: P complex, post-splicing complex. ILS: intron-lariat spliceosome.

Studies in metazoans have shown that the spliceosome can be classified into two types based on the introns they process: the U2-type (major) spliceosome and the U12-type (minor) spliceosome [12]. The major spliceosome comprises five small nuclear ribonucleoproteins (snRNPs)—U1, U2, U4/U6, and U5—along with various non-snRNP factors. Spliceosome-mediated splicing is a complex and dynamic process involving the sequential assembly and disassembly of different snRNPs [13]. This process can be broadly divided into two phases, spliceosome assembly and catalysis, ultimately leading to the formation of a mature transcript (Figure 2). During spliceosome assembly, the process begins with U1 snRNP binding to the 5′ splice site through RNA/RNA base pairing. Simultaneously, splicing factor 1 (SF1) and U2 auxiliary factor (U2AF) recognize and bind to the branch point sequence (BPS) and the polypyrimidine tract (PPT), respectively. U2AF consists of two subunits: U2AF65, which binds the PPT, and U2AF35, which associates with the 3′ splice site. This initial assembly forms the E (early) complex. Next, complex A is established as U2 snRNP binds to the BPS. The U4/U5/U6 tri-snRNP then joins, leading to the formation of complex B. The later dissociation of U1 and U4 triggers structural rearrangements, resulting in an activated complex (B_act_), which then transitions into the catalytically active B* complex. This final complex facilitates intron lariat removal and exon ligation [14]. Furthermore, various non-snRNP protein factors contribute to the splicing process, including the NineTeen Complex (NTC), NTC-associated proteins, serine/arginine (SR)-rich proteins, the REtention and Splicing (RES) complex, and ATPases [15].

## 3. Role of Splicing in Pollen Development

**Figure 3:**
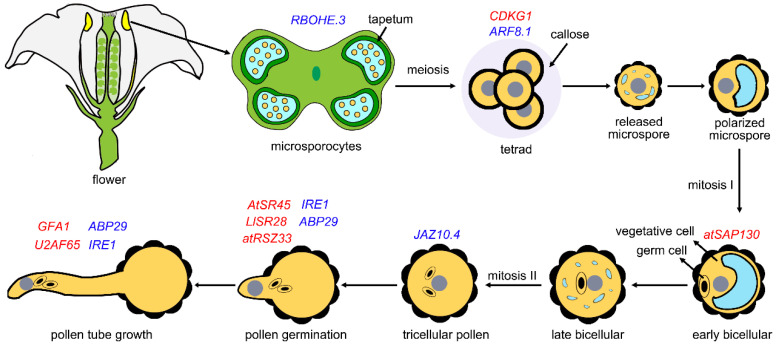
A role of splicing factors in pollen development, germination, and tube growth in *Arabidopsis*. Schematic overview of pollen development, germination, and tube growth in *Arabidopsis*, emphasizing splicing factors crucial for these processes. Pollen development begins within the anther locules, where microsporocytes undergo two meiotic divisions, producing tetrads of four haploid microspores enclosed by callosic cell walls. The tetrads separate as callose is degraded, releasing individual microspores, which then enlarge, polarize, and undergo asymmetric division to form a large vegetative cell and a small generative cell. The generative cell then divides once more to produce two sperm cells, resulting in mature tricellular pollen. Upon germination, pollen grains extend pollen tubes for fertilization. The genes labeled in the figure indicate their functional stages, with red for splicing factors and blue for splicing-related genes.

Alternative splicing increases functional complexity in plants by producing multiple transcript variants from a single gene, thus improving protein diversity [16,17,18,19]. Emerging evidence indicates that alternative splicing isoforms play a role in male gametophyte development (Figure 3, Table 1). *AUXIN RESPONSE FACTOR 8 (ARF8)* produces multiple functionally distinct transcripts through alternative splicing, including *ARF8.1*, *ARF8.2*, and *ARF8.4*, each contributing to different stages and processes of pollen development. The full-length isoform, *ARF8.1*, regulates pollen cell wall formation by directly controlling the expression of key transcription factors—*DEFECTIVE IN TAPETAL DEVELOPMENT (TDF1)*, *ABORTED MICROSPORE (AMS)*, and *MALE STERILITY 188 (MS188*)—in tapetum cells, which are essential for post-meiotic pollen and tapetum development [20]. Anther dehiscence, a crucial step for pollen release and pollination, can be prematurely triggered by the combined action of *ARF8.2* and *ARF8.4. ARF8.2* facilitates anther dehiscence by modulating jasmonic acid (JA) biosynthesis through the regulation of *DEFECTIVE IN ANTHER DEHISCENCE1 (DAD1).* Meanwhile, *ARF8.4* accelerates anther dehiscence by regulating *MYB26*, which influences anther wall lignification. The *ARF8.4* promotes filament elongation by controlling the expression of *INDOLE-3-ACETIC ACID INDUCIBLE19 (AUX/IAA19)*, ensuring optimal pollen positioning for successful pollination [21,22]. Lily 135-ABP is a villin superfamily protein that plays a role in actin cytoskeleton rearrangement. Overexpression of *ABP29*, a splicing variant of *135-ABP*, in lily pollen significantly disrupts actin filament elongation and depolymerization, leading to inhibited pollen germination and pollen tube growth [23]. In *Brassica juncea* var. *tumida*, *T1170* and *T1243* are alternative transcripts of the mitochondrial *T* gene, which is associated with cytoplasmic male sterility. While these isoforms display distinct expression patterns, only *T1243* contributes to the male sterility phenotype [24]. In *Arabidopsis thaliana*, the Jasmonate ZIM-domain 10 (JAZ10) protein functions as a repressor of jasmonate signaling and contains both a Jas domain and a ZIM domain [25]. *JAZ10.4* is a splice variant of *JAZ10* that encodes a protein lacking the Jas domain. Transgenic plants overexpressing *JAZ10.4* show strong JA insensitivity and male sterility due to impaired filament elongation and anther dehiscence [26]. Reactive oxygen species (ROS) signaling plays a crucial role in plant reproduction [27,28]. In *Arabidopsis*, the respiratory-burst oxidase homolog E (RBOHE) is a tapetum-specific NADPH oxidase involved in ROS production, essential for tapetal function and pollen development. *RBOHE* has three splice variants, *RBOHE.1–3*. Overexpression of the *RBOHE.3* splice variant disrupts tapetal ROS homeostasis, accelerates tapetal programmed cell death (PCD), and leads to male sterility [29].

Numerous studies have highlighted the critical role of splicing factors in regulating male gametophyte development (Figure 3, Table 1). U2AF65, a component of U2AF, is essential for recruiting the U2 snRNP complex. It plays a key role in defining 3′ splice sites and regulating alternative splicing [30]. In *Arabidopsis*, two isoforms of *U2AF65*, *U2AF65a* and *U2AF65b*, have been identified. *AtU2AF65b* is involved in the splicing of *FLOWERING LOCUS C* and *ABSCISIC ACID-INSENSITIVE 5*, influencing flowering [31]. The *atu2af65a atu2af65b* double mutants show reduced male transmission and defective pollen tube growth [32]. However, the *AtU2AF65a/b*-spliced genes essential for male gametophyte function and pollen tube growth remain unidentified. Furthermore, a mutation in *GAMETOPHYTIC FACTOR 1* (*GFA1*), which encodes a U5 snRNP-associated protein, disrupts pollen tube growth toward the female gametophyte (FG) [33]. The target genes of GFA1 that regulate pollen tube growth, however, remain unknown.

**Table 1:** RNA splicing factors involved in male gamete formation.

Species	Biological Process	Type of Splicing Factor	Splicing Factor	Targeted Genes	References
*Arabidopsis thaliana*	Male gametophyte transmission, pollen tube growth	Non-snRNP, one subunit of U2AF	U2AF65	Unknown	[32]
*A. thaliana*	Male gametophyte transmission	DEAH-box RNA-dependent ATPase Prp16	CUV	Genes involved in auxin biosynthesis, polar auxin transport, and auxin perception and signaling	[34]
*A. thaliana*	Pollen tube growth	U5 snRNP component	GFA1	Unknown	[33,35,36]
*A. thaliana*	Pollen wall formation	Cyclin-dependent protein kinases (SR protein)	CDKG1	*CalS5*	[37]
*A. thaliana*	Male fertility at modestly ET (elevated temperature)	A major component of the UPR signaling pathway	IRE1	*bZIP60*	[38]
*Lilium longiflorum*	Pollen germination	SR protein	LlSR28	*AtVLN1*	[39]
*A. thaliana*	Pollen germination	SR protein	AtSR45	*AtVLN1*	[39]
*A. thaliana*	Pollen germination, pollen tube growth	SR protein	atRSZ33	Unknown	[40]
*A. thaliana*	Pollen development	A U2 snRNP-associated protein	AtSAP130	*QRT1* and *QRT3*	[41]
*A. thaliana*	Male gametophyte transmission	A U4/U6 snRNP associated protein	RDM16	Unknown	[42]
*A. thaliana*	Male gametophyte development	U5 snRNP component	PRP8A, PRP8B	Unknown	[43]

CYCLIN-DEPENDENT KINASE G1 (CDKG1), an SR motif-containing protein, is recruited to U1 snRNP through its interaction with an arginine/serine-rich splicing factor, facilitating the efficient splicing of *Callose synthase 5* (*CalS5*) [37]. *CalS5* encodes the primary enzyme responsible for synthesizing the callose layer during microspore development [44]. As a result, the *Arabidopsis CDKG1* loss-of-function mutant shows impaired male fertility, characterized by defective pollen wall formation at the tetrad stage [37]. Further studies have established the role of *CDKG1* in meiosis, particularly under high-temperature conditions, where it is essential for homologous chromosome pairing and recombination during male meiosis. Specifically, CDKG1, in complex with CYCLINL, maintains chromosome pairing stability and crossover events, ensuring proper meiotic progression. While *cdkg1* mutants display normal vegetative growth, they become sterile under high temperatures [45]. The findings of a recent study indicate that the long isoform of *CDKG1* (*CDKG1L*) is required for fertility maintenance under high-temperature conditions, whereas the short isoform (*CDKG1S*) cannot rescue fertility in the *cdkg1* mutant [46]. This suggests that the *CDKG1* function is determined not only by its kinase activity but also by the expression and role of its splice isoforms.

*Arabidopsis* SR45 (AtSR45) is an SR-rich protein that regulates the alternative splicing of multiple *SR* gene mRNAs [47]. In the *atsr45* mutant, the full-length transcript of *VILLIN1* increases, while the short transcript decreases, resulting in reduced F-actin levels in hydrated pollen [39]. The lily LlSR28 shares structural similarities with AtSR45. In *atsr45* mutants, pollen germination occurs earlier than in the wild type, whereas *LlSR28* overexpression in *Arabidopsis* completely inhibits pollen germination. These findings suggest that *LlSR28* and *AtSR45* regulate filamentous actin dynamics by modulating the alternative splicing of *VILLIN1*, therefore influencing pollen germination [39]. Similarly, *Arabidopsis* RSZ33 (AtRSZ33) is an SR protein involved in the splicing of *AtSRp30* and *AtSRp34/SR1*. The overexpression of *AtRSZ33* leads to impaired pollen germination [40]. However, the specific target genes regulated by *AtRSZ33* remain unidentified.

## 4. Splicing and Female Gametogenesis

**Figure 4:**
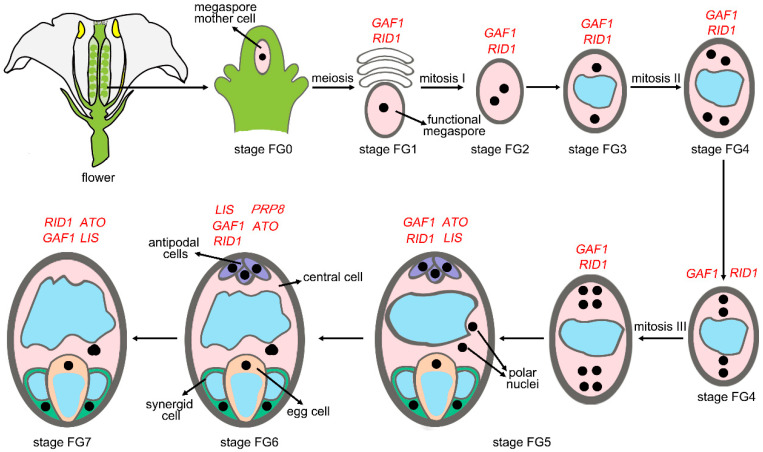
*Arabidopsis* female gametophyte (FG) development highlighting splicing-related factors essential for FG formation. The figure shows *Arabidopsis* female gametophyte (FG) development, highlighting splicing-related factors involved in this process. *Arabidopsis* FG development is categorized into eight stages based on morphological changes. The archesporial cell differentiates into the megaspore mother cell (MMC), which undergoes meiosis to produce a tetrad of haploid megaspores, of which only one survives as the functional megaspore (FM) at stage FG1. The FM undergoes three rounds of mitosis, forming an eight-nucleate coenocyte with four nuclei at each pole separated by a central vacuole. By late FG5, the female gametophyte develops into a seven-celled, eight-nucleate embryo sac with unfused polar nuclei, which fuse at FG6. At FG7, three antipodal cells degenerate, forming a four-celled female gametophyte. At FG8, one synergid cell degrades, resulting in a three-celled female gametophyte. Red-labeled genes represent splicing factors that play roles in different stages of FG development.

Splicing factors also play a crucial role in the normal development of female gametophytes. Mutations in spliceosome-associated genes frequently lead to defects in female gametophyte development (Figure 4, Table 2). For example, GFA1 directly interacts with two U5 snRNP components, AtBRR2 and AtPRP8, as well as the RNA helicase ROOT INITIATION DEFECTIVE 1 (RID1), a homolog of the yeast splicing factor Prp22 in *Arabidopsis* [33,35,36]. *GFA1* and *RID1* are essential for embryo sac development, as they participate in the pre-mRNA splicing of key genes required for female gametophyte formation, including *EMBRYO SAC DEVELOPMENT ARREST 26* and *4*, *FOLYLPOLYGLUTAMATE SYNTHETASE ISOFORM 3*, and *GAST1 PROTEIN HOMOLOG 4* [36,48]. ATROPOS (ATO), a homolog of the yeast pre-mRNA processing factor 9 (PRP9), is a splicing factor associated with U2 snRNP-binding pre-mRNA. Mutation in *ATO* leads to fertility defects, resulting in the production of supernumerary egg cells during female gametophyte development in *Arabidopsis* [49]. *LACHESIS* (*LIS*) encodes an *Arabidopsis* homolog of yeast PRP4, an RNA splicing factor with seven WD40 repeats essential for early spliceosome assembly. In *lis* mutants, female gametophytes show impaired pollen tube attraction, and many accessory cells differentiate into extra egg cells [50,51]. CWC15 is a potential component of the NTC, and its loss of function leads to widespread alterations in splice sites, primarily characterized by increased intron retention. The *cwc15-2* T-DNA insertion mutant displays defective double fertilization, mainly due to significantly reduced female gametophyte transmission efficiency [52]. *Arabidopsis RRC1* encodes a protein similar to the human splicing factor SR140, which is involved in splicing regulation. The null allele *rrc1-4* results in developmental defects in the female gametophyte; however, the specific target genes responsible for this phenotype remain unknown [53].

Many splicing factors are constitutively expressed in plants, and their mutations often lead to defects in the development and function of male and female reproductive organs. *Arabidopsis* SPLICEOSOME-ASSOCIATED PROTEIN 130 (AtSAP130) is a subunit of splicing factor 3b (SF3b), which is essential for pre-spliceosome assembly and pre-mRNA splicing [54]. The RNAi-mediated knockdown of *AtSAP130* results in functional impairments in female reproductive organs and disrupts pollen transition from the microspore to the bicellular stage. AtSAP130 is likely involved in the splicing of *QRT1* and *QRT3*, genes associated with post-tetrad pollen development [41,55]. *Arabidopsis* RDM16 is a U4/U6 snRNP-associated component essential for pre-mRNA splicing. Loss-of-function mutants of *RDM16* show increased intron retention events, reduced viability in both male and female gametophytes, and decreased transmission efficiency through gametes [42]. PRP8 is a core component of the snRNP complex, contributing to the formation of the larger catalytically active spliceosomal B complex [56]. The *Arabidopsis* genome encodes two copies of PRP8, PRP8A and PRP8B, which are involved in the splicing of spliceosome factors and key functional genes required for embryo sac development, including *EMBRYO SAC DEVELOPMENT ARREST 9* (*EAD9*), *EAD30*, *EAD35*, and *MATERNAL EFFECT EMBRYO ARREST 29* (*MEE29*). In the *prp8a prp8b* double mutant, ovules fail to attract pollen tubes, and pollen tube perception of ovular attraction signals is impaired [43].

## 5. Splicing-Regulated Embryo Development

Research has demonstrated that post-transcriptional RNA processing plays a crucial role in embryo development, with mutations affecting splicing leading to abnormal embryogenesis. This highlights the necessity of accurate pre-mRNA splicing for the proper transmission of genetic information during normal plant development (Table 3). *Arabidopsis DEBRANCHING ENZYME 1* (*AtDBR1*) encodes an RNA lariat debranching enzyme, which facilitates the excision of intron lariats during splicing. The mutation of *AtDBR1* causes embryo development to arrest at an early stage [57]. *AtBUD13* encodes an *Arabidopsis* homolog of yeast Bud13, a subunit of the RES complex. Mutations in *AtBUD13* result in early embryonic lethality due to the retention of shorter introns in genes essential for early embryo development [58]. In addition to AtBUD13, GROWTH, DEVELOPMENT AND SPLICING 1 (GDS1), and DAWDLE (DDL) are key components of the RES complex in *Arabidopsis*. Mutations in *GDS1* and *DDL* disrupt the proper splicing of multiple genes involved in cell proliferation and early embryogenesis, leading to developmental defects [59]. Maize *ROUGH ENDOSPERM3* encodes a cofactor of the U2AF35-related protein, influencing alternative splicing and playing a role in embryo and endosperm development as well as their interaction [60]. In *Arabidopsis*, the core splicing factor REPLICATION TERMINATION FACTOR 2 (AtRTF2) is a RING finger-containing protein essential for genome-wide pre-mRNA splicing. Loss of *AtRTF2* function leads to increased intron retention and embryo abortion [61]. LEFKOTHEA (LEFKO), a nuclear-encoded protein, regulates both nuclear and chloroplast pre-mRNA splicing and is crucial for embryogenesis. A *LEFKO* knockout mutation results in embryo lethality due to splicing defects in nuclear and chloroplast gene mRNAs [62]. The direct target genes of LEFKO involved in embryo development regulation remain unidentified. In *Arabidopsis*, AtPRP17, a homolog of the yeast splicing factor PRP17, plays a vital role in embryonic pattern formation. The embryonic lethality observed in *atprp17* mutants is likely due to its involvement in the splicing of genes associated with embryo development [63]. Similarly, JANUS, a homolog of the conserved U2 snRNP assembly factor in yeast and humans, is essential for embryonic pattern formation [64,65]. *Arabidopsis* SM-like (LSM) proteins form two distinct heptameric complexes: LSM1-7 in the cytoplasm and LSM2-8 in the nucleus. The LSM2-8 complex selectively regulates the splicing of specific embryogenesis-related genes, such as *EMBRYO DEFECTIVE 2785 (EMB2785)* and *EMBRYO DEFECTIVE 2016 (EMB2016)*, by stabilizing U6 snRNA. Splicing defects in *lsm8* mutants have been linked to shorter siliques, reduced seed numbers, and seed abortion [66]. SmD3, a core component of the snRNP complex required for pre-mRNA splicing, is encoded by two genes in *Arabidopsis* (*SmD3-a* and *SmD3-b*). The *smd3-b* mutant represents pleiotropic defects, including 10% ovule abortion, while the inability to generate *smd3-a/smd3-b* double mutants suggests that SmD3 is essential for embryogenesis. However, the specific target genes responsible for this phenotype remain unidentified [67].

In higher plants, the splicing of introns in organelle genes relies on a large family of pentatricopeptide repeat (PPR) proteins [78]. These proteins contain 2–30 tandem repeats of a degenerate 31- to 36-amino acid motif, which plays a role in post-transcriptional regulation, including RNA splicing in plant organelles [79]. PPR proteins are classified into two types: the P-type, which contains PPR (P) motifs, and the PLS-type, which includes P motifs along with longer (L) or shorter (S) variants. A subset of P-type PPR proteins has been identified as essential for the splicing of group II introns in organelle genes [80]. MITOCHONDRION-MEDIATED GROWTH DEFECT 1 (MID1) is a P-type PPR protein in *Arabidopsis* that facilitates the splicing of intron 1 in the mitochondrial *NADH dehydrogenase 2* gene by interacting with MITOCHONDRIAL INTRON SF1. Loss-of-function mutations in *MID1* result in arrested embryogenesis [73]. Several P-type PPR proteins in *Arabidopsis*, maize, and rice, including *DEFECTIVE KERNELS and EMPTY PERICARPS*, are involved in the intron splicing of *NADH dehydrogenase* genes encoding subunits of the mitochondrial respiratory chain complex. Mutations in these proteins often lead to mitochondrial dysfunction, adversely affecting embryo and pollen development [69,70,71,72,74,75,76,77,81,82].

Embryo development begins with egg cell fertilization; therefore, defects in female gametophyte development often compromise embryogenesis. For instance, a partial loss-of-function mutation in *GFA1* not only disrupts female gametophyte development but also arrests embryo development before the octant stage [35]. In the *cwc15-2* mutant, impaired female gametophyte function significantly reduces fertilization success. Even when fertilization occurs, abnormal gene expression in *cwc15-2* leads to early embryonic arrest, resulting in an embryonic lethal phenotype [52]. Similarly, the *rrc1-4* mutant shows reduced female gametophyte transmission efficiency while maintaining normal male transmission. The low frequency of *rrc1-4* homozygotes in progeny from self-fertilized *rrc1-4* heterozygotes suggests additional defects in embryogenesis following fertilization [53]. *CLUMSY VEIN* (*CUV*) encodes an *Arabidopsis* ortholog of yeast Prp16, an RNA splicing factor involved in the second-step transesterification reaction during splicing. *CUV* regulates the splicing of genes associated with auxin biosynthesis, transport, perception, and signaling. Phenotypic analysis of *cuv* mutants revealed developmental defects typically linked to auxin, including altered leaf venation, defective gynoecium and stamen development, and arrested embryogenesis [34]. As previously mentioned, the *prp8a prp8b* double mutant disrupts embryo sac attraction for the pollen tube [43], while the *prp8a* mutant alone is embryo-lethal [61,68].

## 6. Conclusions and Future Prospects

This review explores the critical role of pre-mRNA splicing in plant reproductive development, summarizing the functional diversity of various transcripts during reproductive processes and highlighting the essential roles of splicing factors in both female and male gametophytes, as well as embryonic development. These studies demonstrate that the precise regulation of splicing is crucial for the proper progression of plant reproductive development. As a model organism, *Arabidopsis* has laid a crucial foundation for studying pre-mRNA splicing regulation in plant reproductive development. From an evolutionary perspective, the core mechanisms of pre-mRNA splicing, such as the basic composition of the spliceosome, the removal of introns, and splice site recognition, are relatively conserved in higher plants [19,83]. Nevertheless, pre-mRNA splicing regulation networks may vary among different plant species, and this diversity could enable plants to adapt to different environmental conditions and influence their reproductive strategies [19]. Therefore, future research should focus on investigating the role of pre-mRNA splicing in plant reproductive development across more species, exploring potential species-specific pre-mRNA splicing regulatory networks to provide a more comprehensive understanding of plant reproductive development.

Beyond the classic splicing factors, an increasing number of studies show that non-splicing factors also significantly impact plant reproductive development by influencing splicing. For example, *Arabidopsis* INOSITOL-REQUIRING ENZYME 1 (IRE1), a dual-function protein kinase/ribonuclease [84,85], plays a vital role in the splicing of *bZIP60* mRNA at elevated temperatures, which results in reduced male fertility due to interference with pollen coat deposition and normal pollen grain dispersal. Furthermore, the overexpression of *SEC31A*, a gene transcriptionally activated by bZIP60, rescues the temperature-sensitive sterility in *ire1a ire1b* mutants [38]. *SHOOT REDIFFERENTIATION DEFECTIVE 2* (*SRD2*), which encodes a nuclear protein homologous to human SNAP50 that activates snRNA transcription, also influences splicing. Loss of *SRD2* function reduces snRNA levels, impairing splicing and causing reproductive defects, such as failed pollen tube attraction and embryo arrest before the globular stage [86,87,88].

In conclusion, the precise regulation of pre-mRNA splicing, involving both classical and non-classical splicing factors, is essential for plant reproductive development, with these factors influencing splicing through various mechanisms. RNA splicing regulation serves as a crucial mechanism for post-transcriptional gene expression control in plants, offering a faster response to developmental signals compared to the slower processes of transcriptional activation and pre-mRNA accumulation. Many splicing factors play a role in the splicing of key genes involved in plant gametophyte (Table 1 and Table 2) and embryo development (Table 3). However, the mechanisms by which developmental signals—such as hormones and environmental cues—regulate the levels and activities of these splicing factors remain unclear. Investigating how splicing factor levels and activities are modulated by these signals could provide valuable insights into the regulation of splicing during gametophyte and embryo development in plants. Studies have suggested that hormones, epigenetic modifications, and environmental signals influence mRNA splicing [89,90,91,92,93,94,95], but the specific regulation of splicing factors in plant gametophyte and embryo development is yet to be fully explored. Another challenge is identifying the direct mRNA targets of splicing factors involved in these processes. Recent advances in techniques can aid in this task. For example, RNA immunoprecipitation sequencing (RIP-seq) can detect the mRNA targets of splicing factors with RNA-recognition motifs, although it does not reveal binding site information. On the other hand, cross-linking immunoprecipitation (CLIP) coupled with high-throughput sequencing and its variants—individual nucleotide resolution CLIP (iCLIP) and photoactivatable-ribonucleoside-enhanced CLIP (PAR-CLIP)—not only identify the mRNA targets of splicing factors but also provide insights into their binding sites on target mRNAs [96]. Non-coding RNAs (ncRNAs), including microRNA (miRNA), small interfering RNA (siRNA), and long non-coding RNA (lncRNA), play a key role in regulating gene expression both at the transcriptional and post-transcriptional levels [97,98,99]. Multiple studies have shown that ncRNAs are direct targets of splicing factors [100,101,102,103]. Moreover, ncRNAs are involved in plant sexual reproduction and plants’ responses to environmental changes [104,105,106,107,108,109,110,111,112]. Identifying the ncRNA targets of splicing factors could improve our understanding of the role of splicing in plant gametophyte and embryo development.

## Figures and Tables

**Table 2:** RNA splicing factors involved in female gamete formation.

Species	Biological Process	Type of Splicing Factor	Splicing Factor	Targeted Genes	References
*A. thaliana*	Female gametophyte development	DEAH-box RNA-dependent ATPase Prp22	RID1	*EDA26*, *EDA4*, *FGPS3*, *GASA4*	[36,48]
*A. thaliana*	Megagametogenesis	U5 snRNP component	GFA1	*EDA26*, *EDA4*, *FGPS3*, *GASA4*	[33,35,36,49]
*A. thaliana*	Female gametophyte development	U2 snRNP-associated protein	ATO	Unknown	[49]
*A. thaliana*	Supernumerary egg cells, female gametophytes development	U4/U6 snRNP-associated protein	LIS	Unknown	[50,51]
*A. thaliana*	Female gametophyte development	A U2 snRNP-associated protein	AtSAP130	Unknown	[41]
*A. thaliana*	Female gametophyte transmission	A U4/U6 snRNP associated protein	RDM16	Unknown	[42]
*A. thaliana*	A functional redundancy between *PRP8A* and *PRP8B* in female gametophyte development	U5 snRNP component	PRP8A; PRP8B	*EDA9*, *EDA30*, *EDA35*, *MEE29*	[43]
*A. thaliana*	Female gametophyte transmission	Potential component of the NTC	CWC15	Unknown	[52]
*A. thaliana*	Female gametophyte transmission	RS domain (SR-like) protein	RRC1	Unknown	[53]

**Table 3:** RNA splicing factors involved in embryo development.

Species	Biological Process	Type of Splicing Factor	Splicing Factor	Targeted Genes	References
*Zea mays*	Kernel development	RNA lariat debranching enzyme	AtDBR1	Unknown	[57]
*A. thaliana*	Embryogenesis	Nucleus-encoded RNA-binding protein	LEFKO	Unknown	[62]
*A. thaliana*	Early embryogenesis	One composition of the RES complex	AtBUD13	Early embryo developmental genes, e.g., *ZYG1*/*APC11*, *PFI* and *ATML1*	[58]
*A. thaliana*	Early embryogenesis	One composition of the RES complex	DDL	Genes associated with cell proliferation and early embryo development e.g., *AGO10*/*ZLL*, *ATML1*	[59]
*A. thaliana*	Early embryogenesis	One composition of the RES complex	GDS1	Genes associated with cell proliferation and early embryo development e.g., *AGO10*/*ZLL*, *ATML1*	[59]
*A. thaliana*	Embryogenesis	U5 snRNP component	PRP8A	Unknown	[61,68]
*Z. mays*	Kernel development	U2AF35-related protein	Rgh3	Unknown	[60]
*Z. mays*	Kernel development	Mitochondrion-targeted P-type PRP protein	DEK43/DEK41	Mitochondrial *nad4* introns 1 and 3	[69,70]
*Z. mays*	Kernel development	Mitochondria-targeted P-type PPR protein	EMP8	*nad1* intron 4, *nad4* intron 1 and *nad2* intron 1	[71]
*Z. mays*	Kernel development	Mitochondria-targeted P-type PPR protein	DEK35	Mitochondrial *nad4* intron 1	[72]
*A. thaliana*	Embryogenesis	Mitochondria-targeted P-type PPR protein	MID1	Mitochondrial *nad2* intron 1	[73]
*A. thaliana*	Seed development	Mitochondria-targeted P-type PPR protein	OTP43	Mitochondrial *nad1* Intron 1	[74]
*A. thaliana*	Seed development	Mitochondria-targeted P-type PPR protein	SLO3	Mitochondrial *nad7* Intron 2	[75]
*A. thaliana*	Embryogenesis	Rtf2-domain splicing-related protein	AtRTF2	Unknown	[61]
*Oryza sativa*	Grain development	Mitochondrion-targeted P-type PRP protein	RL1	Mitochondrial *nad4* intron 1	[76]
*O. sativa*	Grain development	Nucleolus-localized PPR protein	FLO14/OsNPPR3	*nad 1*–2 and *nad 2*	[77]
*A. thaliana*	Embryogenesis	DEAH-box RNA-dependent ATPase Prp16	CUV	Genes involved in auxin biosynthesis, polar auxin transport, and auxin perception and signaling	[34]
*A. thaliana*	Embryogenesis	U5 snRNP component	GFA1	Unknown	[35]
*A. thaliana*	Embryogenesis	SR protein	AtRSZ33	Unknown	[40]
*A. thaliana*	Embryogenesis	A homolog of the yeast splicing factor PRP17	AtPRP17	Unknown	[63]
*A. thaliana*	Embryonic pattern formation	U2 snRNP assembly factor	JANUS	Unknown	[64,65]
*A. thaliana*	Embryogenesis	U6 SnRNP component (SM-like protein)	LSM	Genes involved in embryo development e.g., *EMB2785*, *EMB2016*	[66]
*A. thaliana*	Embryogenesis	RS domain (SR-like) protein	RRC1	Unknown	[53]
*A. thaliana*	Embryogenesis	SnRNP core protein	SmD3	Unknown	[67]
